# Glucagon-Like Peptide-1 Receptor Agonist Prevented the Progression of Hepatocellular Carcinoma in a Mouse Model of Nonalcoholic Steatohepatitis

**DOI:** 10.3390/ijms21165722

**Published:** 2020-08-10

**Authors:** Motoyasu Kojima, Hirokazu Takahashi, Takuya Kuwashiro, Kenichi Tanaka, Hitoe Mori, Iwata Ozaki, Yoichiro Kitajima, Yayoi Matsuda, Kenji Ashida, Yuichiro Eguchi, Keizo Anzai

**Affiliations:** 1 Division of Metabolism and Endocrinology, Faculty of Medicine, Saga University, Saga 849-8501, Japan; si3219@cc.saga-u.ac.jp (M.K.); takahas2@cc.saga-u.ac.jp (H.T.); f8451@cc.saga-u.ac.jp (T.K.); kensmvc541112@gmail.com (K.T.); sunrisebyebyemoon@yahoo.co.jp (H.M.); ozaki@cc.saga-u.ac.jp (I.O.); f0288@cc.saga-u.ac.jp (Y.K.); matuda-y@med.kyushu-u.ac.jp (Y.M.); ashida@med.kurume-u.ac.jp (K.A.); eguchiyu@cc.saga-u.ac.jp (Y.E.); 2 Liver Center, Saga University Hospital, Faculty of Medicine, Saga University, Saga 849-8501, Japan; 3 Department of Radiology, Eguchi Hospital, Ogi 845-0032, Japan; 4 Department of Medicine and Bioregulatory Science, Graduate School of Medical Sciences, Kyushu University, Fukuoka 812-8582, Japan; 5 Division of Endocrinology and Metabolism, Department of Internal Medicine, Kurume University School of Medicine, Kurume 830-0011, Japan

**Keywords:** liraglutide, hepatocarcinogenesis, ballooning, β-cell, islet, prognosis, liver cancer, NASH, NAFLD

## Abstract

Glucagon-like peptide-1 (GLP-1) receptor agonists are used to treat diabetes, but their effects on nonalcoholic steatohepatitis (NASH) and the development of hepatocellular carcinoma (HCC) remain unclear. In this study, mice with streptozotocin- and high-fat diet-induced diabetes and NASH were subcutaneously treated with liraglutide or saline (control) for 14 weeks. Glycemic control, hepatocarcinogenesis, and liver histology were compared between the groups. Fasting blood glucose levels were significantly lower in the liraglutide group than in the control group (210.0 ± 17.3 mg/dL vs. 601.8 ± 123.6 mg/dL), and fasting insulin levels were significantly increased by liraglutide (0.18 ± 0.06 ng/mL vs. 0.09 ± 0.03 ng/mL). Liraglutide completely suppressed hepatocarcinogenesis, whereas HCC was observed in all control mice (average tumor count, 5.5 ± 3.87; average tumor size, 8.1 ± 5.0 mm). Liraglutide significantly ameliorated steatosis, inflammation, and hepatocyte ballooning of non-tumorous lesions in the liver compared with the control findings, and insulin-positive β-cells were observed in the pancreas in liraglutide-treated mice but not in control mice. In conclusion, liraglutide ameliorated NASH and suppressed hepatocarcinogenesis in diabetic mice. GLP-1 receptor agonists can be used to improve the hepatic outcome of diabetes.

## 1. Introduction

Nonalcoholic fatty liver disease (NAFLD), a hepatic manifestation of metabolic syndrome, affects a large proportion of the global population [1]. It is well known that NAFLD is associated with increased risks of lifestyle-related diseases including type 2 diabetes, cardiovascular disease, and cerebral vessel disease. Nonalcoholic fatty liver disease covers a spectrum of liver diseases that range from benign simple steatosis to hepatic inflammation and fibrosis resulting from nonalcoholic steatohepatitis (NASH), cirrhosis, and hepatocellular carcinoma (HCC) [2,3].

Multiple retrospective studies have evaluated HCC in cryptogenic cirrhosis, supporting the notion that NASH accounts for a large proportion of cryptogenic cirrhosis cases and these lesions can progress to HCC [4]. In these studies, 6%–29% of patients with cryptogenic cirrhosis developed HCC [5,6]. Despite focused efforts and numerous clinical trials, a definitive pharmacological treatment for NASH has not yet been established. Moreover, there is no agent to prevent the development of HCC in patients with NASH.

Recently, studies of incretin-based therapies have focused on both their ability to improve hyperglycemia and their multiple mechanisms of action [7,8]. Incretin hormones stimulate insulin secretion in response to the absorption of orally ingested glucose, thus ensuring an appropriate plasma insulin level to control blood glucose. Glucagon-like peptide-1 (GLP-1) is an incretin hormone with a potent blood glucose-lowering action mediated by its ability to induce insulin secretion and reduce glucagon secretion in a glucose-dependent manner. Furthermore, GLP-1 slows gastrointestinal motility and increases satiety with reduced food intake [9]. Liraglutide is a clinically available GLP-1 receptor agonist. A once-daily subcutaneous formulation was recently licensed in the United States, Europe, and Japan for the treatment of type 2 diabetes [10,11]. Liraglutide ameliorates hepatic steatosis in mice with diet-induced NASH [12]. In humans, previous studies including our clinical trial demonstrated that liraglutide improved the hepatic outcome of NAFLD including serological and histological assessments along with an improvement in glycemic control [13,14]. However, no studies have examined the effects of GLP-1 receptor agonists on the development of HCC in patients with NASH. Therefore, this study aimed to clarify the effects of liraglutide on the progression of NASH and the development of HCC associated with NASH.

## 2. Results

### 2.1. Effect of Liraglutide on Physiology and Biochemistry

**Figure 1:**
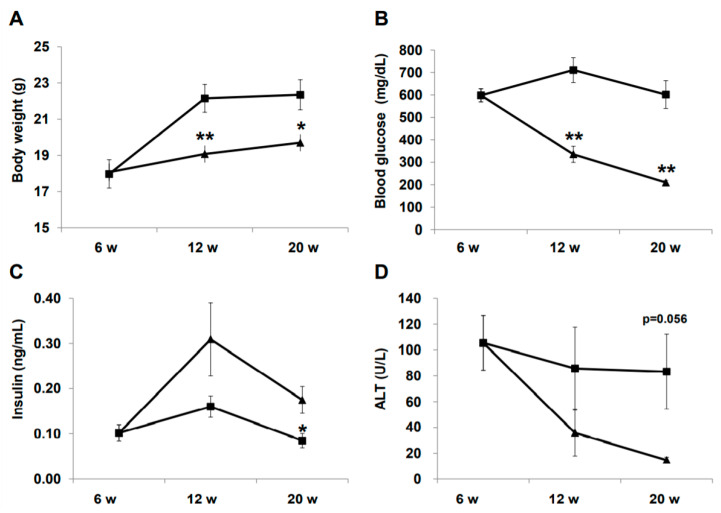
Effect of liraglutide on body weight and biochemical variables. Mice were euthanatized at 6 (*n* = 4 for baseline), 12 (*n* = 6/group), and 20 (*n* = 6/group) weeks (w) of age. Changes in body weight (**A**), blood glucose (**B**), insulin (**C**), and alanine aminotransferase (ALT). Duration of the treatment periods are shown as line graphs (**D**). Data are presented as the mean, and error bars represent the standard deviation. Triangle: liraglutide (LIRA)-treated mice; square: control mice (CTR). * *p* < 0.05 and ** *p* < 0.01 vs. CTR.

Changes in physical and biochemical variables during treatment are summarized in Figure 1. Body weight was significantly lower in the liraglutide (LIRA) group than in the control (CTR) group (Figure 1A). The LIRA group exhibited lower fasting blood glucose (210.0 ± 17.3 mg/dL vs. 601.8 ± 123.6 mg/dL; *p* = 0.0008) and higher fasting insulin levels (0.18 ± 0.06 ng/mL vs. 0.09 ± 0.03 ng/mL; *p* = 0.035) at 20 weeks of age (Figure 1B,C). Aminotransferase (ALT) levels tended to be lower in the LIRA group than in the CTR group (Figure 1D).

### 2.2. Development of HCC

**Figure 2:**
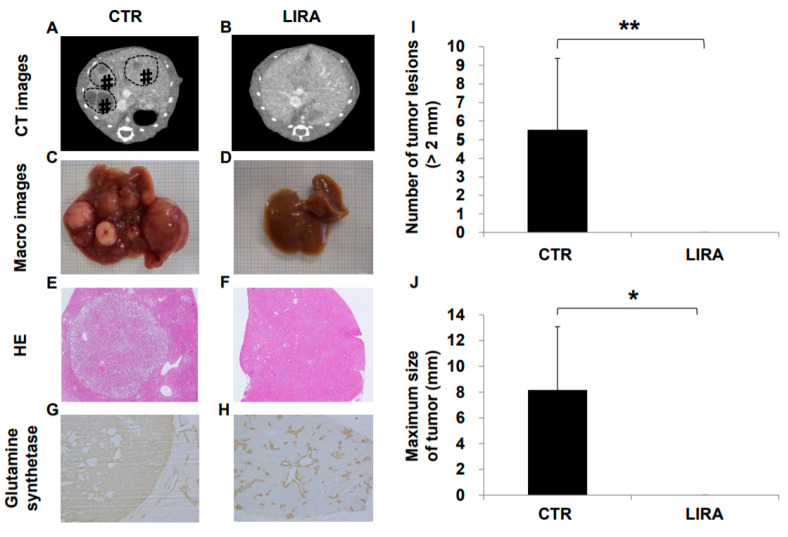
Effect of liraglutide on hepatocarcinogenesis. Representative contrast-enhanced computed tomography images of control mice (CTR) (**A**) and liraglutide-treated mice (LIRA) (**B**). # and dotted line circles denote the HCC lesions. Macroscopic image of the liver surface (CTR (**C**); LIRA (**D**)). Histological images with hematoxylin and eosin staining in the CTR (**E**) and LIRA groups (**F**). Anti-glutamine synthetase staining of CTR (CTR (**G**); LIRA (**H**)). Total number of tumor lesions (**I**) and maximum size (**J**) of tumors in the liver were compared between the groups (*n* = 6/group). All images and histological samples were obtained from mice at 20 weeks of age. * *p* < 0.05 and ** *p* < 0.01 vs. CTR.

Hepatocarcinogenesis was compared between the LIRA and CTR groups at 20 weeks of age (Figure 2). Hepatocellular carcinoma was observed in all CTR mice on computer tomography (CT), whereas no LIRA mice displayed tumor lesions (Figure 2A,B). In addition, in the gross anatomical evaluation (Figure 2C,D), we did not observe HCC in any mice in the LIRA group, whereas CTR mice displayed noticeable HCC lesions (mean lesion number, 5.5 ± 3.9, *p* < 0.01; mean lesion size, 8.1 ± 5.0 mm, *p* < 0.05 (Figure 2I,J). In hematoxylin and eosin (HE)-stained tumor specimens, well to moderate differentiated HCC with capsules was observed in the CTR group (Figure 2E,F). Hepatocellular carcinoma was also detected in the CTR group on the basis of anti-glutamine synthetase positivity (Figure 2G). Non-hepatocellular carcinoma cells around the central vein were also positive for anti-glutamine synthetase in both groups (Figure 2G,H).

### 2.3. Histological Evaluation of NASH

**Figure 3:**
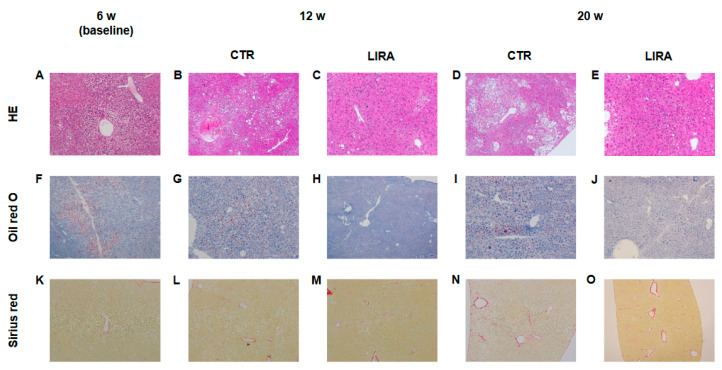
Representative histological images of non-tumorous lesions at 20 weeks (w) of age. Hematoxylin and eosin(**A**–**E**), staining (HE). Oil red O staining (**F**–**J**). Sirius red staining (**K**–**O**). LIRA: liraglutide-treated mice; CTR: control mice

**Figure 4:**
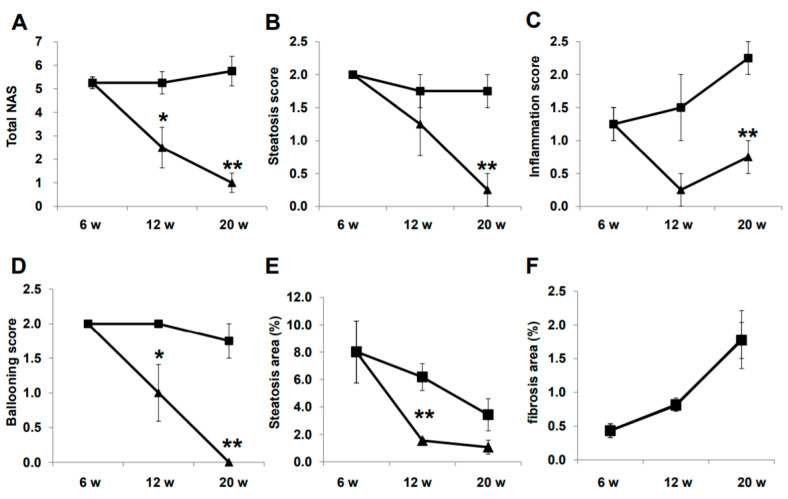
Histological evaluation of non-tumorous lesions. Mice were euthanatized at 6 (*n* = 4 for baseline), 12 (*n* = 6/group), and 20 (*n* = 6/group) weeks (w) of age. Changes in total nonalcoholic fatty liver disease activity score (NAS) (**A**), steatosis score (**B**), inflammation score (**C**), ballooning score (**D**), fat deposition areas evaluated using oil red O-stained samples (**E**), and fibrotic areas evaluated using Sirius red-stained samples (**F**) are presented as line graphs. Data are presented as the mean, and error bars represent the standard deviation. Triangle: liraglutide-treated mice (LIRA); square: control mice (CTR). * *p* < 0.05 and ** *p* < 0.01 vs. CTR.

Representative histological images of non-HCC lesions are shown in Figure 3. At baseline, moderate steatosis was observed in HE- and oil red O-stained specimens in the CTR group, whereas steatosis was ameliorated in the LIRA group (Figure 3A–J). Regarding the changes in steatosis, hepatocyte ballooning was improved in the LIRA group, whereas no changes versus baseline were noted in the CTR group. Liver fibrosis was mild at baseline and slightly aggravated in both groups (Figure 3K–O). The histological results for non-HCC lesions are summarized in Figure 4. The LIRA group displayed a significantly lower NAFLD activity score (NAS) (Figure 4A), including decreases in the steatosis (Figure 4B), inflammation (Figure 4C), and ballooning scores (Figure 4D), whereas no change in NAS or its component scores were identified in the CTR group. Fat deposition was significantly decreased in the LIRA group at 12 weeks of age (Figure 4E). Liver fibrosis was aggravated in both groups (Figure 4F).

### 2.4. Histological Evaluation of Pancreatic β-Cells

**Figure 5:**
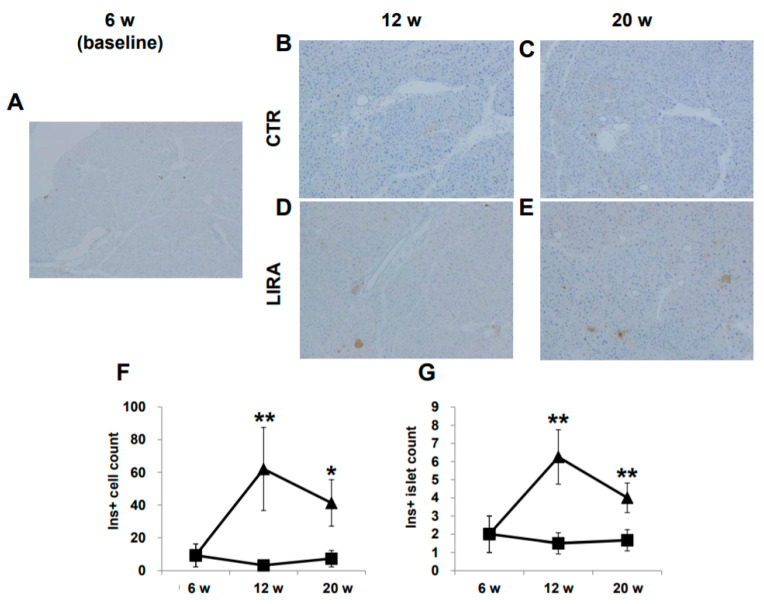
Histological images of the pancreas. Mice were euthanatized at 6 (*n* = 4 for baseline), 12 (*n* = 6/group), and 20 (*n* = 6/group) weeks (w) of age. There were no anti-insulin antibody-stained cells at baseline (**A**), 12 w (**B**), and 20 w (**C**) in the control mice (CTR), whereas islets with insulin-positive (Ins+) cells were observed in the liraglutide-treated mice (LIRA) (**D**,**E**). The counts of Ins + cells (**F**) and islets with Ins+ cells (**G**) were compared between the groups. Data are presented as the mean, and error bars represent the standard deviation. Triangle: LIRA (*n* = 4–6); square: CTR (*n* = 4–6). * *p* < 0.05 and ** *p* < 0.01 vs. CTR.

Representative images of pancreatic β-cells are shown in Figure 5A–E. β-cells were completely ablated by streptozotocin (STZ) treatment, and no insulin-positive cells were detected at baseline. Insulin-positive cells were observed in the LIRA group at 12 and 20 weeks of age, whereas insulin staining was negative in CTR mice. The number of insulin-positive β-cells and the number of islets containing insulin-positive β-cells were significantly higher in the LIRA group (Figure 5F,G).

### 2.5. Development of HCC in the Delayed Therapy

**Figure 6:**
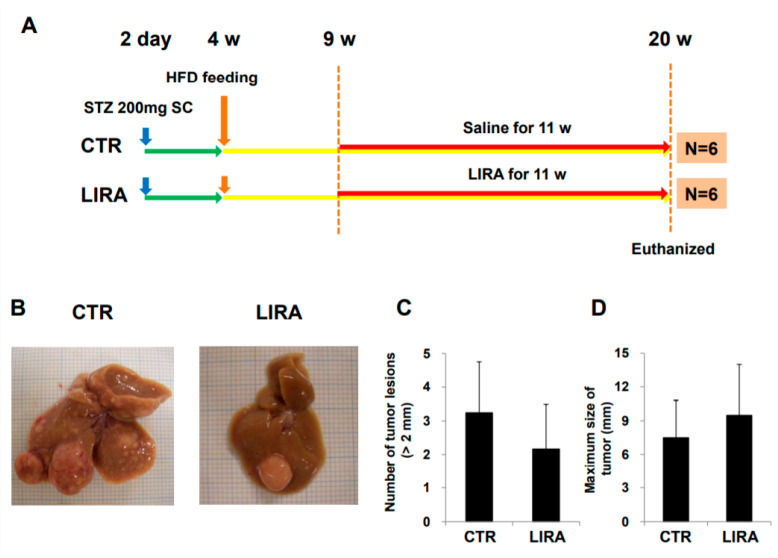
Effect of delayed treatment with liraglutide on hepatocarcinogenesis. (**A**) Experimental design and protocol. A total of 200 µg streptozotocin was injected into 15-day-old male mice at 2 days after birth followed by feeding with a high-fat diet after 4 weeks (w) of age. Mice were killed at 20 w of age (*n* = 6/group). Mice were treated with liraglutide (LIRA) or saline (CTR) for 11 w starting at 9 w of age and were killed at 20 w of age. (**B**) Macroscopic image of the liver surface. The total number of tumor lesions (**C**) and maximum size (**D**) of tumors in the liver were compared between the groups. CTR: control mice; LIRA: liraglutide-treated mice; STZ: streptozotocin; SC: subcutaneous injection; HFD: high-fat diet.

**Figure 7:**
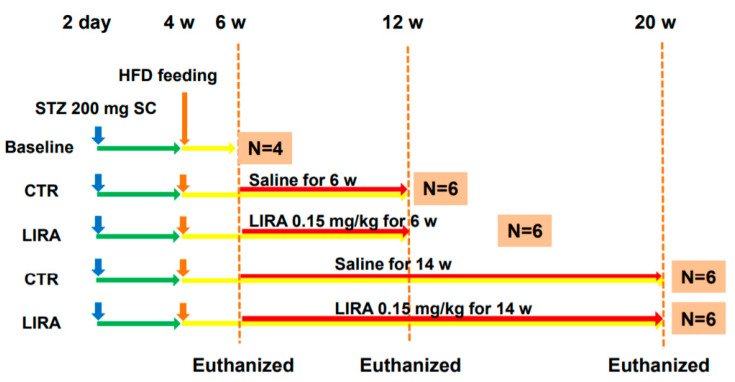
Experimental design and protocol. A total of 200 µg streptozotocin was injected into 15-day-old male mice at 2 days after birth followed by feeding with a high-fat diet after 4 weeks (w) of age. Mice were killed at 6 (*n* = 4 for baseline), 12 (*n* = 6/group), and 20 (*n* = 6/group) w of age. Mice were treated with liraglutide or saline for 6 w or 14 w until death. CTR: control mice; LIRA: liraglutide-treated (0.15 mg/kg by subcutaneous injection) mice; STZ: streptozotocin; SC: subcutaneous injection; HFD: high-fat diet.

After delaying treatment until 9 weeks of age (Figure 6A; treatment was delayed for 3 weeks from the original experimental design shown in Figure 7), hepatocarcinogenesis was evaluated in the gross anatomy of mice. HCC nodules were observed in the livers of mice in both groups (Figure 6B). There was no significant difference in the number of tumor lesions (LIRA, 2.2 ± 1.3; CTR, 3.3 ± 1.5; *p* = 0. 26) or maximum tumor size (LIRA, 9.5 ± 4.5 mm; CTR, 7.5 ± 3.3 mm; *p* = 0.47) between the LIRA and CTR groups (Figure 6C,D).

## 3. Discussion

Liraglutide improves hepatic outcomes, including the histological findings of NASH and glycemic control in rodents and humans [12,13,14], but its effect on hepatocarcinogenesis associated with NASH is unclear. Our current study clarified the suppressive effect of liraglutide on hepatocarcinogenesis in a mouse model of diabetes and NASH. Surprisingly, the anti-hepatocarcinogenesis effect was conclusive, and liraglutide completely suppressed hepatocarcinogenesis in mice treated with liraglutide for 14 weeks. Moreover, the current study confirmed that liraglutide ameliorates the histological findings of NASH in mice with diabetes and NASH.

Hyperglycemia caused by β-cell ablation resulting from STZ treatment and high-fat diet feeding, concomitant with NASH and hepatocarcinogenesis at a relatively young age, is a unique feature of this mouse model [15]. Hyperglycemia mediates oxidative stress; Protein kinase C-delta expression; production of reactive oxygen species and advanced glycation end-products, which induce hepatic inflammation; DNA damage; and carcinogenesis [16,17,18]. Therefore, the anti-hepatocarcinogenesis effect of liraglutide in the current study could be explained by the amelioration of hyperglycemia. In the current study, liraglutide increased pancreatic β-cells in STZ-treated mice (Figure 5). As a pharmacological therapy for diabetes in humans, GLP-1 analog has a major physiological role in the amelioration of glycemic control in pancreatic β-cells. Glucagon-like peptide-1 enhances glucose-stimulated insulin secretion from β-cells [19], stimulates proliferation and neogenesis, and inhibits β-cell apoptosis [20]. Trans-differentiation of α-cells and pancreatic ductal cells to β-cells in rodents treated with GLP-1 has been reported [21,22]. These multiple effects of liraglutide on β-cells might have contributed to the increased β-cell numbers, recovering insulin secretion, and ameliorating the hyperglycemia observed in the current study. Recent studies demonstrated that different hypoglycemic agents, including sodium glucose transporter inhibitor 2 inhibitors and dipeptidyl peptidase 4 inhibitors, suppressed the development of HCC following the improvement of glycemic control in a similar mouse model as that used in the present study [23,24]. This suggests that the amelioration of hyperglycemia could be a critical mechanism in preventing hepatocarcinogenesis, independent of the treatment modality. Hypoinsulinemia is another unique characteristic of the mouse model used in the current study. Interestingly, hypoinsulinemia, similar to hyperinsulinemia, independently aggravates fatty liver through fatty acid transport protein 2 (FATP2) or FATP5 expression [25]. In the current study, we observed increased serum insulin levels and pancreatic β-cell counts in mice treated with liraglutide. Taken together, the improvement in glucose homeostasis induced by liraglutide could contribute to its suppressive effects on hepatocarcinogenesis.

Conversely, the mouse model used in the current study does not exhibit obesity and insulin resistance, which generally characterize type 2 diabetes and NASH. According to epidemiological studies, increased body weight is associated with an increased risk of liver cancer and increased death rates for liver cancer [26,27]. Obese people generally have insulin resistance, and this is often associated with elevated levels of circulating insulin [28]. However, in the current study, obesity and insulin resistance were not observed (Figure 7). Recently, lean and non-obese NAFLD has been recognized, especially in Asia, and is considered to have less severe hepatic fibrosis [29,30]. Despite a lower BMI, visceral obesity in cases of “lean” NAFLD causes insulin resistance, which could be less severe than that in “obese” NAFLD and might result in a less severe hepatic outcomes [30]. However, advanced liver fibrosis is observed at a certain prevalence in lean NAFLD [29,30], which suggests that there might be a potential mechanism that promotes liver fibrosis in lean NAFLD. It is reported that pancreatic β-cell function is impaired in NASH [31]. Moreover, Musso et al. demonstrated the association between the severity of hepatic fibrosis and pancreatic β-cell dysfunction in NAFLD [32]. Racial differences in β-cell function are well known, and β-cell function is lower in Asians [33]. Therefore, the mouse model used in the current study and the obtained findings could represent an aspect of the disease phenotype of lean and non-obese NASH, and liraglutide might prevent disease progression and hepatocarcinogenesis in NAFLD, including lean and non-obese NASH.

Some recently reported in vivo and in vitro data supported a direct mechanism for the effects of GLP-1 receptor agonists on the liver and on hepatocytes. Glucagon-like peptide-1 receptors have been identified on both murine and human hepatocytes [34,35,36,37]. Glucagon-like peptide-1 receptor agonists activate fatty acid oxidation and reduce lipogenesis by modulating lipid transport, AMP-activated protein kinase (AMPK), β-oxidation, and insulin signaling, which have been suggested as critical mechanisms of the pathogenesis of NAFLD/NASH [35,36,37]. AMP-activated protein kinase is also associated with the prevention of cancer development [38]. AMP-activated protein kinase activation causes cell cycle arrest, which is associated with the stabilization of p53 and the cyclin-dependent kinase inhibitors p21 and p27 [39,40]. AMP-activated protein kinase inhibits the mechanistic target of rapamycin complex-1 [41]. Zhou et al. reported that injection of exenatide, a GLP-1 agonist, significantly reduced diethylnitrosamine-induced hepatocellular carcinoma in both low-fat low-carbohydrate- and high-fat high-carbohydrate-fed mice, which suggests that exenatide prevented hepatocarcinogenesis independent of obesity and NASH [42]. In this study, the preventive effect of exenatide was associated with the high expression of GLP-1 receptor and the activation of cyclic AMP (cAMP) and protein kinase A (PKA). Moreover, exenatide downregulated signal transducer and activator of transcription 3 (STAT3), which suppressed multiple STAT3-targeted genes including *MYC* and *CCND1*. Activation of cAMP and PKA by exenatide was confirmed in an in vitro study using hepatoma, colon cancer, and breast cancer cells [43,44]. cAMP–PKA–STAT3 signaling is also associated with the anti-inflammatory effect of GLP-1. Li et al. reported that liraglutide increased the ratio of M2/M1 Kupffer cells in the liver of high fat diet-fed mice [45]. In the same report, in vitro studies using primary Kupffer cells found that liraglutide treatment modulated M2-like activation in Kupffer cells via the cAMP–PKA–STAT3 signaling pathway. Another in vitro study using a human macrophage cell line, RAW264.7, elucidated that GLP-1 agonists increase M2 macrophage-related markers and the secretion of the anti-inflammatory cytokine Interleukin-10 and revealed that GLP-1 agonist induces macrophages to develop the M2 phenotype [46]. Hepatocyte apoptosis is a key mediator of liver injury, inflammation, and fibrosis in chronic liver diseases including NAFLD [47]. Hepatocyte apoptosis is mediated by lipid accumulation, cellular stress derived from oxidative metabolic disorder, cytokine alterations, and mitochondrial dysfunction [48]. Hepatocyte apoptosis positively correlates with liver disease severity and hepatic fibrosis [49]. Recently, it was reported that GLP-1 decreased miR-23a expression and Bcl-2-associated X protein pathway, and attenuated hepatic apoptosis in HepG2 cells cultured in high glucose conditions [50]. Another study elucidated that GLP-1 enhances ATP binding cassette subfamily a member 1-dependent cholesterol efflux and reduces hepatocyte apoptosis [51]. Taken together, these direct effects of GLP-1 agonist on hepatocytes, Kupffer cells, and hepatic stellate cells, as well as the improvement in glucose homeostasis, could have contributed to the prevention of NASH progression and hepatocarcinogenesis in the current study. Conversely, a 3-week delay in liraglutide treatment negated these therapeutic effects in the current study (Figure 6). Earlier treatment could be important in controlling NASH and hepatocarcinogenesis in clinical settings.

Glucagon-like peptide-1 agonist also affects collagen synthesis by human stellate cells (HSC). Liraglutide inhibits the activation of HSCs and α-smooth muscle actin expression by blocking the p38 mitogen-activated protein kinase signaling pathway in vitro [52]. Moreover, liraglutide ameliorated liver fibrosis with an improvement in hepatic microvascular function in rats treated with carbon tetrachloride [53]. However, in our current study, there was no significant difference in liver fibrosis between the groups (Figure 1). Differences in the animal model and in the mechanism of liver fibrosis of the individual model might modulate the effects of liraglutide. In essence, the liver fibrosis of the mouse model used in the current study is mild to moderate compared with that in carbon tetrachloride-induced rodent models [15]. Indeed, aggravation of liver fibrosis was limited at 20 weeks of age, as shown in Figure 4, whereby the effect of liraglutide on liver fibrosis might be difficult to determine. Further research is required to confirm the effects of liraglutide on liver fibrosis, especially in NASH. However, according to our results, the effects of liraglutide on the prevention of hepatocarcinogenesis might be independent of its effects on liver fibrosis in the mouse model of diabetes.

There are several limitations in the current study. Although the effects of liraglutide on NASH and hepatocarcinogenesis were, at least partly, demonstrated in this study, the underlying molecular mechanism remains unclear and should be addressed in the future. Because the related evidence was not enough to calculate the sample size when we conducted the experiment, the number of mice was small. Further studies including a greater number of mice and different NASH/HCC models should be conducted.

## 4. Materials and Methods

### 4.1. Animal Model

Model mice of NASH-HCC were established according to the protocol of Fujii et al. [15] (so-called STAM mice). In brief, pathogen-free pregnant 15-day-old C57BL/6 mice were purchased from Charles River Laboratories (Kanagawa, Japan). NASH and NASH-HCC were induced in male mice through a single subcutaneous injection of 200 µg streptozotocin (STZ; Sigma-Aldrich, St. Louis, MO, USA) at 2 days after birth and through feeding with a 32% fat diet (60 kcal% fat; CLEA, Tokyo, Japan) ad libitum after 4 weeks of age. This mouse model progresses from NAFLD to NASH at 8 weeks of age and develops to HCC at 16 weeks of age [15]. The animals had free access to water and food, and they were maintained in a specific pathogen-free facility under controlled temperature (23 ± 2 °C), humidity (50 ± 20%), and light conditions (12 h light/12 h dark). All animal protocols and procedures were approved by the institutional review board (Stelic Institute, MNP011-1209-2, Apr 3 2013) and were performed in accordance with the “Guide for the Care and Use of Laboratory Animals” prepared by the National Academy of Sciences and published by the National Institutes of Health (NIH publication 86-23, revised 1985).

### 4.2. Experimental Design

The experimental design and protocol are shown in Figure 7. Mice for the baseline analysis were euthanatized at 6 weeks of age. Mice were divided into two groups and treated once daily with subcutaneous saline (control, CTR group) or 0.15 mg/kg liraglutide (LIRA group) starting at 6 weeks of age. Mice were euthanized at 12 (treated for 6 weeks) or 20 weeks (treated for 14 weeks) of age. Blood samples were obtained from the right atrium via cardiac puncture, and their livers and pancreas were excised. To investigate the effect of delayed treatment with liraglutide on the progression of NASH, a separate cohort of mice was treated with either saline or 0.15 mg/kg liraglutide starting at 9 weeks of age and euthanized at 20 weeks of age (Figure 6A).

### 4.3. Biochemistry Analysis

Serum blood glucose, alanine aminotransferase (ALT), and triglyceride levels were measured using a dry chemistry analyzer (FUJI DRI-CHEM 7000, Fujifilm, Tokyo, Japan). Plasma insulin concentrations were quantified using an Ultra-Sensitive Mouse Insulin ELISA Kit (Morinaga Institute of Biological Science, Yokohama, Japan).

### 4.4. Histological Analysis

Liver samples were fixed in Bouin’s fluid, embedded in paraffin or cryopreserved in an optimal cutting temperature compound (Sakura Finetek, Tokyo, Japan), and snap-frozen in liquid nitrogen. Paraffin sections were stained with a solution of Lillie-Mayer’s hematoxylin (Muto Pure Chemicals, Tokyo, Japan) and eosin (HE; Wako, Osaka, Japan) or Sirius red. To visualize macro- and micro-vesicular fat, we fixed the cryosections with 4% Paraformaldehyd-phosphate buffered saline and stained them with oil red O (Sigma-Aldrich). Using HE-stained samples, we calculated the NAFLD activity score (NAS) by combining the grades of steatosis (0–3), inflammation (0–3), and hepatocyte ballooning (0–2) [54]. The areas of liver fibrosis and steatosis in Sirius red- and oil red O-stained samples were measured using ImageJ software (version 1.52r, 26 October 2019, (https://imagej.nih.gov/ij/download.html). For immunohistochemistry, the cryosections were stained overnight at 4 °C with optimal dilutions of anti-glutamine synthetase (Cat# MAB302, Chemicon International, Temecula, CA, USA) to confirm the tumor lesion as HCC [55,56] and anti-insulin antibodies (Cat# MAB1417, R&D Systems, Minneapolis, MN, USA) to identify pancreatic β-cells. The substrate reaction, after incubation with appropriate secondary antibodies, was performed using 3′,3′-diaminobenzidine solution (Nichirei, Tokyo, Japan). Insulin-positive cells were counted on slides of the entire pancreas of individual mice.

### 4.5. Imaging Analysis Using Computer Tomography

Computer tomography (CT) was performed in mice at 20 weeks of age (2 days before the start of treatment). The mice were mounted on a holder and placed in the X-ray CT system (LCT-200, Aloka, Japan) under pentobarbital sodium (Kyoritu Seiyaku, Japan) anesthesia. Contrast reagent (Iopamiron, Bayer HealthCare, Germany) was injected intravenously to obtain contrast-enhanced images.

### 4.6. Statistical Analysis

Continuous variables are summarized as the mean ± standard deviation. An unpaired *t*-test was used to analyze continuous ordinal data for two qualitative variables. Differences were considered significant at *p* < 0.05. All analyses were performed using IBM SPSS (Version 21.0; SPSS Inc., Tokyo, Japan).

## 5. Conclusions

In conclusion, the administration of GLP-1 receptor agonists prevents the development of HCC in mice with diabetes and NASH. Clinically, GLP-1 receptor agonists could represent a treatment option to improve the hepatic outcome of patients with diabetes.
